# An *in-silico* benchmark for the tricuspid heart valve – Geometry, finite element mesh, Abaqus simulation, and result data set

**DOI:** 10.1016/j.dib.2021.107664

**Published:** 2021-12-02

**Authors:** Devin W. Laurence, Chung-Hao Lee, Emily L. Johnson, Ming-Chen Hsu

**Affiliations:** a School of Aerospace and Mechanical Engineering, The University of Oklahoma, 865 Asp Ave., Felgar Hall 212, Norman, OK 73019, USA; b Department of Aerospace and Mechanical Engineering, University of Notre Dame, Notre Dame, IN 46556, USA; c Department of Mechanical Engineering, Iowa State University, Ames, IA 50011, USA

**Keywords:** Tricuspid valve, Finite element simulations, *In-Silico* benchmark, Leaflet-to-leaflet contact, Material nonlinearity

## Abstract

This article provides Abaqus input files and user subroutines for performing finite element simulations of the tricuspid heart valve with an idealized geometry. Additional post-processing steps to obtain a ParaView visualization file (*.vtk) of the deformed geometry are also provided to allow the readers to use the included ParaView state file (*.pvsm) for customizable visualization and evaluation of the simulation results. We expect this *first-of-its-kind in-silico* benchmark dataset will facilitate user-friendly simulations considering material nonlinearity, leaflet-to-leaflet contact, and large deformations. Additionally, the information included herein can be used to rapidly evaluate other novel *in-silico* approaches developed for simulating cardiac valve function. The benchmark can be expanded to consider more complex features of the tricuspid valve function, such as the dynamic annulus motion or the time-varying transvalvular pressure. Interested readers are referred to the companion article (Johnson et al., 2021) for an example application of this *in-silico* tool for isogeometric analysis of tricuspid valves.

## Specifications Table

**Table:** 

Subject	Bioengineering
Specific subject areas	Computational Mechanics and Mechanical Engineering
Type of data	Geometry file, finite element mesh input, user material subroutine, finite element simulation output, post processing scripts, processed simulation results
How data were acquired	All simulation inputs and results were generated and produced in-house.
Data format	Idealized Valve Geometry (*.igs, *.3dm, *.obj) Abaqus Input Files – Finite Element Model (*.inp) Abaqus Output Files – Simulation Results (*.odb) FORTRAN Source Code – VUMAT (*.for) C Source Code (*.C) Matlab Source Code (*.m) Batch File (*.bat) Text Files (*.txt) ParaView Visualization Toolkit File (*.vtk) ParaView State File (*.pvsm)
Parameters for data collection	Abaqus simulations were performed following the benchmarking problem outlined in [1], with specific details described in Appendix C.
Description of data collection	Data were collected by running the Abaqus simulation using the *.inp and *.for files included herein.
Data source location	Institution: The University of Oklahoma City/State: Norman, Oklahoma Country: United States of America
Data accessibility	All data is available with the article.
Related research article	E. L. Johnson, D. W. Laurence, F. Xu, C. E. Crisp, A. Mir, H. M. Burkhart, C.-H. Lee, and M.-C. Hsu, Parameterization, geometric modeling, and isogeometric analysis of tricuspid valves, Comput. Methods Appl. Mech. Engrg., 384 (2021) 113960. https://doi.org/10.1016/j.cma.2021.113960

## Value of the Data

• The data in this article provide the idealized tricuspid valve geometry and Abaqus input files used in Ref. [1]. The provided information is readily used for finite element simulations, and the predictions can be compared with our provided closed tricuspid valve geometry.
• The information and data provided within this article will assist researchers in the areas of cardiovascular heart valve biomechanics and computational mechanics who seek to develop novel *in-silico* techniques. The user-friendly nature of our shared information allows for others to quickly and easily replicate our simulation results for verification of their implemented shell formulation for modeling tricuspid heart valves.
• We envision that the contents of this article can be used as an *in-silico* benchmark platform for the tricuspid valve, the other three cardiac valves, and bioprosthetic valve devices. Specifically, the provided benchmark tricuspid valve model geometry allows for *in-silico* simulations to capture valve closing behaviors that involve material nonlinearity, leaflet-to-leaflet contact, and large deformations.
• The additional post-processing routine provided in this article can also assist researchers in more customizable visualization and evaluation of the simulation results in an open-source software—ParaView.

## 1 Data Description

This article provides Abaqus (Dassault Systémes Simulia Corp., Johnson, Rhode Island [2]) input files and user subroutines to perform finite element simulations of an idealized tricuspid valve geometry. Additional post-processing information is included to help the readers transform the Abaqus simulation output into a ParaView (Kitware Inc., Clifton Park, New York [3]) *.vtk file that can be used to evaluate the simulation predictions. The files and data associated with this article have been arranged into five directories (see Table 1).

**Table 1:** Summary and description of the data files associated with this article.

Directory	File Name	Description
Idealized_Geometry	Idealized_TV.igs	Used for creating the finite element mesh
	Idealized_TV.3dm	Additional format for the idealized geometry
	Idealized_TV.obj	Additional format for the idealized geometry

Abaqus_Simulation_Files	00_Run_Model.inp	Master input file
	01_Model_node.inp	Contains all nodes
	02_Model_elem_leaflet.inp	Defines leaflet elements
	03_Model_elem_chord.inp	Defines chordae elements
	04_Model_section.inp	Constructs model sections
	05_Model_amp.inp	Defines loading condition amplitude
	06_Model_material.inp	Defines the material models
	07_Model_boundary.inp	Defines boundary conditions
	08_Model_output.inp	User-defined outputs
	vumat_combined.for	Subroutine for material models
	00_Run_Model.odb	Raw Abaqus output

Post_Processing	ODB_process.bat	Transforms the .odb to .txt files
	process_elem.C	Creates the element .txt files
	process_node.C	Creates the node .txt files
	make_VTK.m	Creates the ParaView .vtk files

Text_Files	00_Run_Model_elem_FrameX.txt	Element information for simulation frame X
	00_Run_Model_node_FrameX.txt	Node information for simulation frame X

ParaView_Visualization	00_Run_Model_FrameX.vtk	Visualization file for simulation frame X
	Visualize.pvsm	ParaView state file for visualization

### 1.1 Idealized geometry

**Fig. 1:**
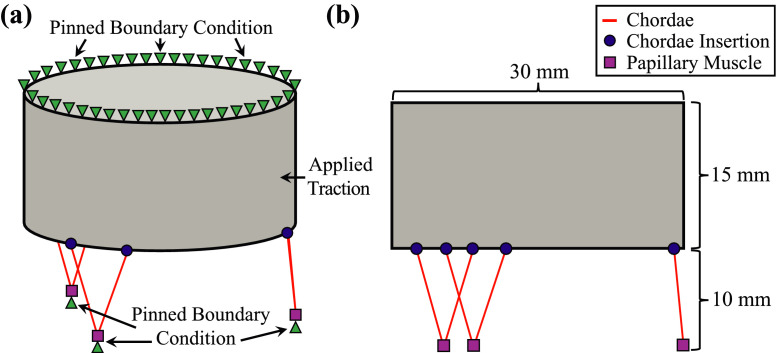
The idealized tricuspid valve geometry: **(a)** isometric view with labeled boundary and loading conditions and **(b)** side view showing the dimensions as well as the papillary muscle and chordae insertion locations.

**Fig. 2:**
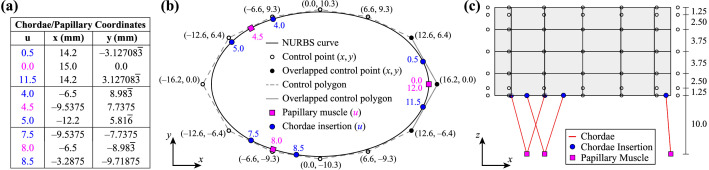
Definition of the idealized tricuspid valve geometry: **(a)** Table of chordae insertions and papillary muscle parametric coordinates (u) and corresponding x- and y-coordinates, **(b)** top view showing the oval-shaped valve curve, control points, and papillary muscle and chordae insertion locations, and **(c)** side view showing the distance between control points. All x-, y-, and z-coordinates and distances are in millimeters (mm). The knot vectors are {−3,−2,−1,0,1,2,3,4,5,6,7,8,9,10,11,12,13,14,15} and {0,0,0,0,1,2,3,4,4,4,4} in the circumferential and vertical directions, respectively.

The surface of the idealized geometry (Fig. 1) is constructed with an oval-shaped annulus that is defined by a periodic, cubic non-uniform rational B-spline (NURBS) curve. The valve surface is defined by 12 × 4 cubic B-spline elements, and each chordae is defined by a two-node, linear B-spline element (Fig. 2). The idealized tricuspid valve geometry files are provided in multiple formats: Idealized_TV.3dm (Rhino 3D), Idealized_TV.obj (Wavefront), and Idealized_TV.igs (IGES). Please note that when exporting the geometry into the IGES format via Rhino, the periodic surface is converted into a non-periodic surface. The IGES file can be opened in the Abaqus/CAE environment to prepare the finite element mesh. Details regarding the idealized geometry creation and finite element mesh preparation are found in *Tricuspid Valve Model Geometry and Mesh*.

### 1.2 Abaqus simulation files

The nine Abaqus *.inp files associated with this article (see Table 1) have been constructed to allow for easy manipulation of the simulation input. First, the master input file (00_Run_Model.inp) lists the *.inp files to be used in the simulation. Therein, the node input file (01_Model_node.inp), two element input files (02_Model_elem_leaflet.inp, and 03_Model_elem_chord.inp) describe the idealized tricuspid valve geometry (Fig. 1), which are used in the section input file (04_Model_section.inp) to construct the sections and define node/element sets associated with the prescribed boundary and loading conditions. Next, a linear amplitude profile for the applied surface pressure is defined (05_Model_amp.inp) and the material properties are assigned to the geometry sections (06_Model_material.inp). Finally, the displacement boundary conditions and simulation step are defined (07_Model_boundary.inp), followed by the nodal and elemental result output (08_Model_output.inp). Each of these *.inp files includes several Abaqus keywords (beginning with a single asterisk) that may be viewed as the building blocks of the simulation. More information, regarding specific keywords or data lines, can be found within the Abaqus Keyword Reference Guide [4].

**Fig. 3:**
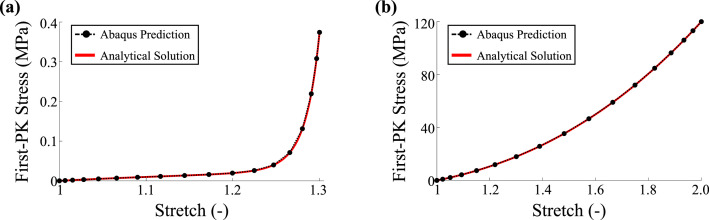
Verification of the VUMAT subroutines: **(a)** the tricuspid valve leaflet subjected to equibiaxial tension and **(b)** the tricuspid valve chordae under uniaxial stretching.

The Fortran source code (user_combined.for) provides the VUMAT user subroutines for the materials models of both the tricuspid valve leaflet and the chordae. The subroutine first checks the material model name provided by the Abaqus .inp file for the given element: (1) tricuspid valve leaflet material model (for S4 shell elements, Fig. 3a) and (2) tricuspid valve chordae material model (for T3D2 truss elements, Fig. 3b) and then calculates the material stresses at each time step for the respective element type. Details regarding the VUMAT subroutine can be found in the Abaqus User’s Manual [5].

The output database file (00_Run_Model.odb) generated by Abaqus is a binary file that summarizes the results for each simulation “frame”, or user-defined output time point. Unlike the other files herein, 00_Run_Model.odb is written in a binary file format that is only readable in the Abaqus/CAE environment or requires an additional interpreter for converting the data into an ASCII text file. Once opened with Abaqus, the user can visualize the simulation outputs (e.g., stress or strain) as defined in 08_Model_output.inp. It should be noted that several other files will be generated by the Abaqus software once the simulation is executed. However, we are only concerned with the finite element predictions of the material deformations and stresses (i.e., *.odb) for the purpose of this article. A complete description of these extra files can be found in the Abaqus User’s Manual [6].

### 1.3 Post processing

The *.bat file and two *.C scripts included in this directory construct the ASCII *.txt files that summarize the finite element predictions for each simulation frame. When the batch file (ODB_process.bat) is submitted to the command prompt, the two *.C files are first transformed into *.exe files usable by Abaqus. Next, the element information (i.e., process_elem.C) and node information (i.e., process_node.C) are extracted from the *.odb file and written to their respective text files in the Text_Files directory.

The additional Matlab script (make_VTK.m) included in this directory uses the generated *.txt files to create ParaView *.vtk files in the ParaView_Visualization directory. This script has been written in a flexible manner to allow for the user to define a range of simulation frames for processing. Additionally, the file names and directories used by the script can be updated to work with any folder structure beyond what has been provided with this article.

### 1.4 Text files

This directory contains all of the *.txt files generated by the post processing scripts (see *Post Processing*). Each *.txt file associated with element data contains 11 columns of data – Column 1: indication of Cauchy “Stress” or logarithmic “Strain” data, Column 2: element number, Column 3: integration point, Column 4: number of stress components (i.e., 4), Columns 5–8: stress or strain components, Column 9: maximum in-plane principal stress or strain, Column 10: minimum in-plane principal stress or strain, and Column 11: von Mises stress (not applicable for “Strain” data lines). On the other hand, the node *.txt files contain 13 columns of data – Column 1: node number, Columns 2-4: *x-y-z* coordinates, Columns 5–7: displacement components, Columns 8–10: reaction force components, and Columns 11–13: reaction moment components.

### 1.5 ParaView visualization

**Fig. 4:**
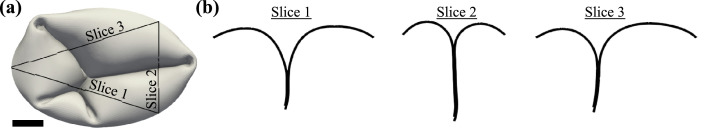
ParaView visualization of the Abaqus simulation result: **(a)** top view with labeled slice views and **(b)** various slice views. Scale bar =5 mm.

This directory includes the *.vtk files generated by the post processing scripts (see *Post Processing*) for each simulation frame. The ParaView state file (Visualize.pvsm) provided with this article enables the user to visualize the prepared *.vtk file (Fig. 4a) and quickly perform more in-depth analysis of the simulations by a series of cut views (Fig. 4b). Detailed instructions are provided in *Visualization of the simulation results*.

## 2 Experimental Design, Materials and Methods

### 2.1 Tricuspid valve model geometry and mesh

The idealized tricuspid valve geometry (Idealized_TV.igs) shown in Fig. 1a was prepared using B-spline curves and surfaces within the Rhino software (Robert McNeel & Associates, Seattle, Washington [7]). Specific details necessary to reproduce the idealized geometry are provided in Fig. 11 of the companion article [1]. In brief, the tricuspid valve annulus was represented using an oval shape with major and minor axis dimensions of 30 mm and 20 mm, respectively (Fig. 1b). A uniform leaflet height of 15 mm was used to generate the leaflet surfaces, and the papillary muscles were located 10 mm below the leaflet free edge (Fig. 1c). These dimensions were selected to provide a realistic representation of typical porcine tricuspid valve dimensions [1], [8].

To create the mesh of the tricuspid valve geometry, the leaflet surface was discretized into 18,960 shell elements (S4) using an approximate global seed size of 0.25 mm, while each chordae was represented using one 3D truss element (T3D2). We assumed a uniform leaflet thickness of 0.396 mm [9] and a uniform chordae radius of 0.23 mm [9]. The chordae insertions into the leaflets were represented using shared nodes between the chordae and leaflet elements (see Fig. 1).

### 2.2 Material models

In accordance with the previous cardiac valve biomechanics studies (e.g., [10]), we assume the tricuspid valve leaflets are transversely isotropic an incompressible solids [11]. Hence, the tricuspid valve leaflets were modeled using the Lee-Sacks hyperelastic strain energy density function [12].

(1) $$W=\frac{c_{0}}{2}\left(I_{1}-3\right)+\frac{c_{1}}{2}\left(\delta e^{c_{2}{\left(I_{1}-3\right)}^{2}}+\left(1-\delta \right)e^{c_{3}{\left(I_{4}-1\right)}^{2}}-1\right)-p\left(J-1\right).$$

Herein, W is the strain energy density, $I_{1}=\text{tr}C$ and $I_{4}=m\cdot (Cm)$ are the first invariant and fourth psuedo-invariant of the right Cauchy-Green deformation tensor $C=F^{T}F$, F is the deformation gradient, m is the unit vector that defines the collagen fiber orientation, p is the penalty parameter to enforce tissue incompressibility (i.e., J=detF=1), $c_{0}=10$ kPa, $c_{1}=0.209$ kPa, $c_{2}=9.046$, and $c_{3}$ are the material parameters [1], and δ represents the collagen fiber alignment where δ=0 indicates perfectly aligned collagen fibers (i.e., anisotropic material) and δ=1 indicates randomly oriented collagen fibers (i.e., isotropic material) [12]. For the purpose of this tricuspid valve *in-silico* benchmark, we only considered the *isotropic* component of Eq.  (1) (i.e., δ=1) so the $I_{4}$ term in Eq.  (1) vanishes. However, the collagen fiber architecture information, such as that acquired via polarized spatial frequency domain imaging [13], could be incorporated into the material model, rendering the *anisotropic* material behaviors typically observed in planar biaxial mechanical characterizations. $p=2\left(\partial W/\partial I_{1}\right)/{\left(C^{-1}\right)}_{33}$ is analytically determined by considering the tissue incompressibility and assuming a plane stress condition.

The chordae were modeled as a Saint Venant–Kirchhoff material without the Poisson effect:

(2) $$S_{11}=\left(\lambda +2\mu \right)E_{11}$$

where $S_{11}$ is the 1−1 component of the second Piola–Kirchhoff stress, $E_{11}=\left({F_{11}}^{2}-1\right)/2$ is the 1−1 component of the Green-Lagrange strain, and λ and μ are the Lamé constants. Considering a Young’s modulus E=40 MPa and a Poisson’s ratio ν=0 [9], the Lamé constants are λ=0 and μ=20 MPa.

The implementations of the material models within the Abaqus VUMAT subroutine were verified using the analytical solutions for a chordae undergoing uniaxial tension (Eq.  (2)) and TV leaflet undergoing equibiaxial tensions. The analytical stresses for the leaflets were computed using

(3) $$S=2\frac{\partial W}{\partial C}=-pC^{-1}+2\frac{\partial W}{\partial I_{1}}I+2\frac{\partial W}{\partial I_{4}}m⊗m$$

considering the strain energy density function in Eq.  (1), while the analytical stresses for the chordae were computed using the stress-strain relationship in Eq.  (2). The agreement between the finite element and analytical stresses for both the TV leaflet (Fig. 3a) and the chordae (Fig. 3b) indicated successful implementation of the material models in the VUMAT subroutine.

### 2.3 Boundary and loading conditions

Three simplifications were made regarding the boundary and loading conditions. First, for simplicity we assumed the tricuspid valve annulus and chordae tendineae were pinned throughout the simulation, although these are dynamic structures during *in vivo* cardiac function [14], [15]. Second, the time-varying transvalvular pressure typically experienced by the tricuspid valve leaflets (see Fig. 5 of [16]) was simplified as a linear increase to 25 mmHg over 0.005 s that was maintained for a total simulation time of 0.4 s. The simulation was performed using Abaqus Explicit dynamics with a viscous damping pressure of $5\times 10^{-6}$ MPa·s and a maximum time step of $1\times 10^{-6}$  s in adaptive time-stepping to ensure proper convergence.

### 2.4 Visualization of the simulation results

Additional steps were performed to visualize the simulation results stored in the Abaqus *.odb binary file. First, the results from each output frame were converted to an ASCII .txt file by running the ODB_process.bat batch file in the command prompt. Next, the ParaView .vtk file for each frame was generated by running the CreateVTK.m MATLAB script. The resulting .vtk file can be opened using the ParaView Visualize.pvsm state file for customizable visualization (see Fig. 4) of the simulation results. Additionally, the .vtk files can be opened using a text editor software (e.g., Notepad++ [17]) to view the node and element information for a specified simulation frame.

It is important to note the localized buckling of the tricuspid valve leaflets in this *in-silico* benchmark may result in multiple simulation solutions. Therefore, the reader may observe differences in the predicted tricuspid valve configuration when comparing their results with those provided in this article. This could stem from differences between computers, variations in domain discretization (not employed herein), possible machine errors, or the use of different simulation approaches. We have not yet observed this phenomenon in our *in-silico* benchmark. However, the companion article [1], which employed isogeometric analysis, demonstrated multiple solution configurations when considering various levels of mesh refinement.

## CRediT authorship contribution statement

**Devin W. Laurence:** Conceptualization, Methodology, Software, Investigation, Visualization, Writing – original draft. **Chung-Hao Lee:** Conceptualization, Methodology, Supervision, Funding acquisition, Writing – review & editing. **Emily L. Johnson:** Conceptualization, Methodology, Writing – review & editing. **Ming-Chen Hsu:** Conceptualization, Funding acquisition, Writing – review & editing.

## Declaration of Competing Interest

The authors declare that they have no known competing financial interests or personal relationships which have, or could be perceived to have, influenced the work reported in this article.

## Appendix A Supplementary materials

Supplementary Data S1 Supplementary Raw Research Data. This is open data under the CC BY license http://creativecommons.org/licenses/by/4.0/ Supplementary Data S1
